# The Aspergillus fumigatus Mismatch Repair *MSH2* Homolog Is Important for Virulence and Azole Resistance

**DOI:** 10.1128/mSphere.00416-19

**Published:** 2019-08-07

**Authors:** Thaila Fernanda dos Reis, Lilian Pereira Silva, Patrícia Alves de Castro, Rafaela Andrade do Carmo, Marjorie Mendes Marini, José Franco da Silveira, Beatriz Henriques Ferreira, Fernando Rodrigues, Abigail Lee Lind, Antonis Rokas, Gustavo H. Goldman

**Affiliations:** a Faculdade de Ciências Farmacêuticas de Ribeirão Preto, Universidade de São Paulo, Ribeirão Preto, Brazil; b Departamento de Microbiologia, Imunologia e Parasitologia, Escola Paulista de Medicina, Universidade Federal de São Paulo, São Paulo, Brazil; c Life and Health Sciences Research Institute (ICVS), School of Medicine, University of Minho, Braga, Portugal; d ICVS/3B’s—PT Government Associate Laboratory, Braga/Guimarães, Portugal; e Department of Biological Sciences, Vanderbilt University, Nashville, Tennessee, USA; Carnegie Mellon University

**Keywords:** *Aspergillus fumigatus*, DNA repair, MSH2, azole resistance, virulence

## Abstract

The genetic stability of every living organism depends on accurate DNA replication and repair systems. Here, we investigated the Aspergillus fumigatus *MSH2* mismatch repair (MMR) gene MshA and how it impacts virulence and the evolution of azole resistance. We examined *mshA* gene variation in 62 environmental and clinical A. fumigatus strains. We have observed 12 strains with variants (18.2%), and 8 strains among them showed missense variants. We demonstrated that A. fumigatus *mshA* null mutants are haploid and have conserved karyotypes with discrete gross chromosomal rearrangements. The Δ*mshA* strains are not sensitive to several DNA-damaging agents. The lack of *mshA* caused a significant reduction of virulence of A. fumigatus in a neutropenic murine model of invasive pulmonary aspergillosis and in the invertebrate alternative model Galleria mellonella. Wild-type and Δ*mshA* populations did not show any significant changes in drug resistance acquisition after they were transferred 10 times in minimal medium in the absence of any stress. However, these populations rapidly acquired virulence in the Δ*mshA* background and high levels of resistance to posaconazole in the presence of this drug (at least 200-fold-higher levels of resistance than those derived from the wild-type strain). Taken together, these results suggest that genetic instability caused by Δ*mshA* mutations can confer an adaptive advantage, mainly increasing posaconazole resistance and virulence acquisition.

**IMPORTANCE** Invasive aspergillosis (IA) has emerged as one of the most common life-threatening fungal diseases in immunocompromised patients, with mortality rates as high as 90%. Systemic fungal infections such as IA are usually treated with triazoles; however, epidemiological research has shown that the prevalence of azole-resistant Aspergillus fumigatus isolates has increased significantly over the last decade. There is very little information about the importance of genomic stability for A. fumigatus population structure, azole resistance, and virulence. Here, we decided to investigate whether the mismatch repair system could influence A. fumigatus azole resistance and virulence, focusing on one of the components of this system, *MSH2*. Although the mutation frequency of *mshA* (the A. fumigatus *MSH2* homologue) is low in environmental and clinical isolates, our results indicate that loss of *mshA* function can provide increased azole resistance and virulence when selected for. These results demonstrate the importance of genetic instability in A. fumigatus as a possible mechanism of evolving azole resistance and establishing fitness in the host.

## Abstract (precis)

Invasive aspergillosis (IA) has emerged as one of the most common life-threatening fungal diseases in immunocompromised patients, with mortality rates as high as 90%. Systemic fungal infections such as IA are usually treated with triazoles; however, epidemiological research has shown that the prevalence of azole-resistant Aspergillus fumigatus isolates has increased significantly over the last decade. There is very little information about the importance of genomic stability for A. fumigatus population structure, azole resistance, and virulence. Here, we decided to investigate whether the mismatch repair system could influence A. fumigatus azole resistance and virulence, focusing on one of the components of this system, *MSH2*. Although the mutation frequency of *mshA* (the A. fumigatus *MSH2* homologue) is low in environmental and clinical isolates, our results indicate that loss of *mshA* function can provide increased azole resistance and virulence when selected for. These results demonstrate the importance of genetic instability in A. fumigatus as a possible mechanism of evolving azole resistance and establishing fitness in the host.

## INTRODUCTION

Aspergillus fumigatus causes several clinical diseases, including the life-threatening disease invasive pulmonary aspergillosis (IA), with high mortality rates in neutropenic patients (1–4). Systemic fungal infections such as IA are usually treated with antifungal drugs such as polyenes, azoles, and echinocandins, the first two targeting cell membrane ergosterol biosynthesis and the latter perturbing the biosynthesis of the cell wall polysaccharide glucan (5). Echinocandins represent a relatively new class of antifungal agents which act by noncompetitively, inhibiting the cell wall enzyme β-1,3-glucan synthase and therefore impairing fungal cell wall biosynthesis and integrity (6). Echinocandins present fungistatic activity against *Aspergillus* spp. and are regularly used as second-line therapy for treatment of IA (7). Triazole resistance is becoming a major concern in A. fumigatus, and it has already been observed in six of the seven continents. Resistance can evolve through exposure to azole compounds during azole therapy or in the environment (8). Mutations in the *cyp51A* gene, whose protein product is targeted by azoles, represent the main mechanism of resistance (9); however, other known and unknown resistance mechanisms may be present (9).

Genetic stability is essential for the survival and maintenance of living organisms. DNA mismatch repair (MMR) is a system for recognizing and repairing erroneous insertion, deletion, and misincorporation of bases that can arise during DNA replication and recombination, as well as repairing some forms of DNA damage (10, 11). Evolution of fungal drug resistance has been associated with MMR (12–15). Drug resistance arises more rapidly in Candida albicans strains lacking MMR proteins or proteins central to double-strand break repair (12). Comparison of mutation frequencies in deletion strains of eight MMR genes in C. neoformans showed that the loss of three of them, *MSH2*, *MLH1*, and *PMS1*, results in an increase in mutation rates, allowing rapid generation of resistance to antifungal agents (13). Healey et al. (14) have previously demonstrated that strains carrying mutations in Candida glabrata MMR gene MSH2 exhibit a higher propensity for breaking through antifungal treatment *in vitro* and in mouse models of colonization. The mutator genotype was not associated with increases in fluconazole resistance in C. glabrata isolates of a French cohort of patients harboring low rates of resistance but was instead found to be related to rare and specific genotypes (16). Absence of azole or echinocandin resistance was observed in C. glabrata isolates in India despite a background prevalence of MMR-defective strains (17). In China, C. glabrata bloodstream isolates showed that *PDR1* multidrug transporter polymorphisms were associated with acquisition of fluconazole resistance whereas *MSH2* polymorphisms were not correlated with fluconazole resistance (18). It is possible that MMR represents a partial explanation for the elevated rates of triazole and multidrug resistance associated with C. glabrata (14, 15).

We previously investigated the A. fumigatus AtmA (Ataxia-telangiectasia mutated) and AtrA kinases and how they impact virulence and the evolution of azole resistance (19). We have observed that genetic instability caused by Δ*atmA* and Δ*atrA* mutations can confer an adaptive advantage mainly in the intensity of voriconazole resistance acquisition but not in virulence. Here, we investigated the influence of Msh2, a protein that binds to DNA mismatches initiating the MMR, on A. fumigatus azole resistance. We observed a low level of polymorphism in the *mshA* gene (here referred to as the A. fumigatus *MSH2* homologue) in A. fumigatus in clinical and environmental isolates. We also constructed A. fumigatus *mshA* null mutants and demonstrated that the lack of mutations in *mshA* could influence virulence in both a neutropenic murine model of invasive pulmonary aspergillosis and in the moth Galleria mellonella as an alternative animal model. We also demonstrated that different populations of the null *mshA* mutants grown through 10 sequential mitotic passages can evolve virulence attributes in the G. mellonella model of infection. We also observed that MshA plays an important role in the development of increased azole resistance in A. fumigatus.

## RESULTS

### A. fumigatus *MSH2* homolog (*mshA*) has low levels of genetic polymorphism in clinical and environmental isolates.

**FIG 1:**
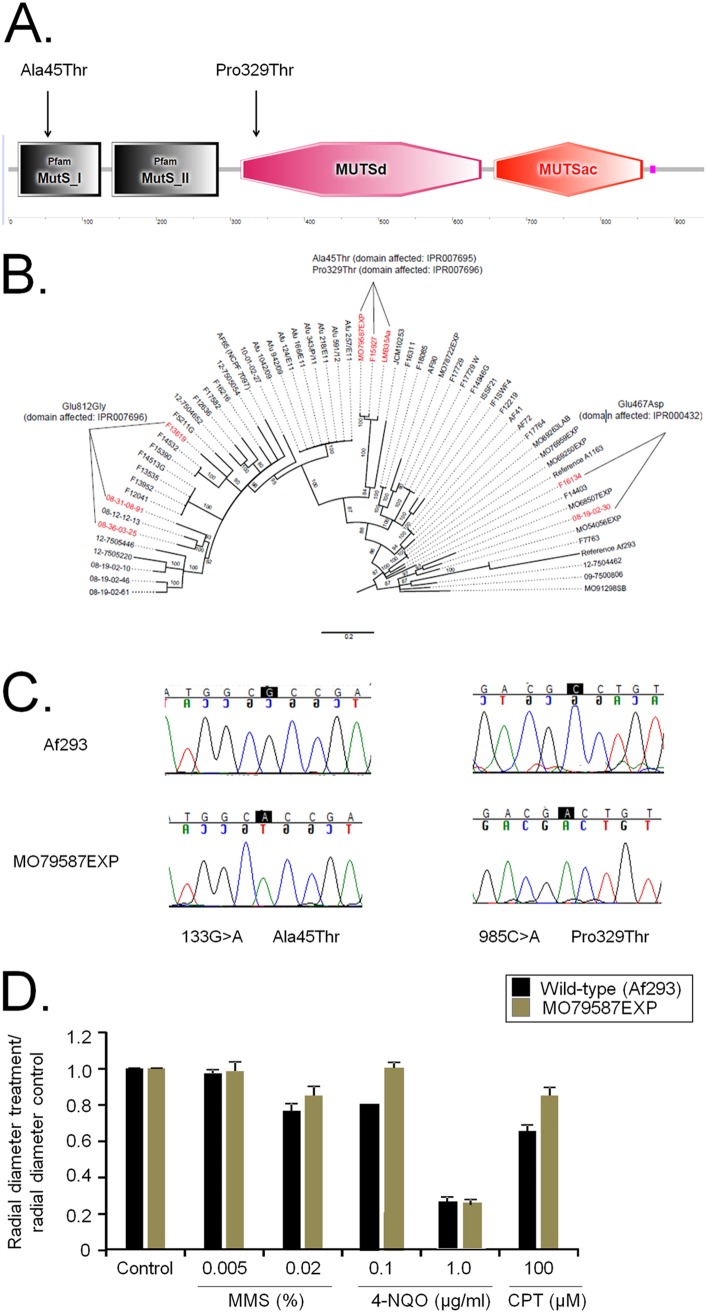
Genetic polymorphism of *mshA* in 62 A. fumigatus clinical and environmental isolates. (A) Organization of MshA as predicted using the SMART interface (http://smart.embl-heidelberg.de/). MshA has the following domains: MutS_I (N terminus; 4.3E−17; IPR007695), amino acids 13 to 125; MutS_II (connector domain; 1E−20; IPR007860), amino acids 140 to 284; MuTSd (core; DNA-binding domain of DNA mismatch repair MUTS family; 5.68E−105; IPR007696), amino acids 314 to 639; MUTSac (C terminus; ATPase domain of DNA mismatch repair MUTS family; 1.61E−118; IPR000432), amino acids 656 to 857. (B) Phylogenetic tree for the *mshA* gene showing the single nucleotide polymorphisms (SNPs) in amino acid predictions. (C) Chromatograms (from Sanger method for DNA sequencing) of a small region of the *mshA* gene from strains Af293 and MO79587EXP showing the SNPs. (D) A. fumigatus Af293 and MO79587EXP conidia (1 × 10^4^) were inoculated on MM with different drug concentrations. Plates were incubated for 5 days at 37°C. The results were expressed as averages of radial diameter of the treatment area divided by the radial diameter of the control from three independent experiments ± standard deviations. MMS, methyl methanesulfonate; 4-NQO, 4-nitroquinoline oxide; CPT, camptothecin.

Saccharomyces cerevisiae Msh2p was used as a query to identify the orthologue A. fumigatus 3g09850 (Afu3g09850; here named MshA) (E value, 00; identity, 46.7%; similarity, 64.7%). A. fumigatus *mshA* encodes a putative protein with 940 amino acids and a molecular weight of 105.3 kDa. The organization of protein domains analyzed by using the SMART interface (http://smart.embl-heidelberg.de/) showed that the structure and organization of the A. fumigatus protein are highly conserved compared to those of S. cerevisiae Msh2p (Fig. 1A). MshA has the following domains: MutS_I (N terminus; 4.3E−17; IPR007695), amino acids 13 to 125; MutS_II (connector domain; 1E−20; IPR007860), amino acids 140 to 284; MuTSd (core; DNA-binding domain of DNA mismatch repair MUTS family; 5.68E−105; IPR007696), amino acids 314 to 639; MUTSac (C terminus; ATPase domain of DNA mismatch repair MUTS family; 1.61E−118; IPR000432), amino acids 656 to 857.

We examined *mshA* variation in 62 environmental and clinical A. fumigatus strains (for a description of these strains, see reference 20). Determination of genome-wide variation relative to the Af293 strain revealed 12 strains (18.2%) with 4 variants in the 5′ and 3′ untranslated regions (UTR), 13 strains with variants in the coding region (8 missense and 4 synonymous variants), and 7 strains with intergenic variants (Table 1; see also Table S1 in the supplemental material). Eight strains (12.1%) showed missense variants: 3 strains with Glu812Gly changes (domain affected, IPR007696), 3 strains with Ala45Thr and Pro329Thr changes (domains affected, IPR007695 and IPR007696, respectively), and 2 strains with Glu467Asp (domain affected, IPR000432) (Fig. 1A) (Table 1; see also Table S1).

**TABLE 1:** Missense mutations observed in A. fumigatus environmental and clinical isolates

Strain containing variant	Position of change on chromosome	Amino acid affected
MO79587EXP	133 G>A	Ala45Thr
MO79587EXP	985 C>A	Pro329Thr
F16134	1401 G>C	Glu467Asp
08-31-08-91	2435 A>G	Glu812Gly

10.1128/mSphere.00416-19.3 TABLE S1 Identified SNPs for *mshA* in the environmental and clinical isolates. Download Table S1, XLS file, 0.03 MB. Copyright © 2019 dos Reis et al. 2019 dos Reis et al. This content is distributed under the terms of the Creative Commons Attribution 4.0 International license.

The *mshA* gene does not have a high missense substitution rate across strains. We counted all missense mutations per gene across all strains, calculated the number of missense SNPs per base pair per gene, and ranked them. There were 8,860 protein-coding genes in which we detected at least one missense mutation, and *mshA* ranked as the 7,795th most commonly missense-substituted gene. Looking at missense SNPs in the MO79587EXP strain, *mshA* is ranked as the 6,086th most commonly missense-substituted gene in this strain.

The MO79587EXP strain does not have an elevated rate of nonsynonymous substitution relative to other strains, but it is part of a group of 3 strains that are more divergent from Af293 than the remaining strains. These three strains have about twice as many variants on average than the strains that are less divergent from Af293, and this is true for all classes of variants, including missense and synonymous variants.

We also looked at the mutation rates in *msh6* (Afu4g08300) and *rad51* (Afu1g10410) to see whether *mshA* had an unusual rate of substitution for a DNA repair gene. The *msh6* gene ranked as the 3,556th most frequently missense-substituted gene (of a total of 8,860), and 59 strains had a missense substitution in this gene. However, 2 missense substitutions in this gene are shared across >50 strains, so it is not likely that this gene is frequently mutating but rather that the Af293 allele is uncommon in this set of strains. There was only 1 observed missense variant in the *rad51* gene in one strain. These findings suggest that *mshA* is not an uncommonly frequent target of genetic change in A. fumigatus.

Although we were able to download all the genome sequences from public databases, despite several efforts and requests for access to these strains, we were unable to obtain them. Thus, we conducted our analyses only on the MO79587EXP clinical isolate, which was previously characterized by us (20). Sanger sequencing validated the two point mutations in this strain in comparison to the Af293 strain (Fig. 1A and C). The MO79587EXP strain showed the same resistance to methyl methanesulfonate (MMS), 4-nitroquinoline oxide (4-NQO), and camptothecin (CPT), posaconazole, voriconazole, and caspofungin as the wild-type strain (Fig. 1D and data not shown).

### Molecular and functional characterization of A. fumigatus MSH2 orthologue MshA.

**FIG 2:**
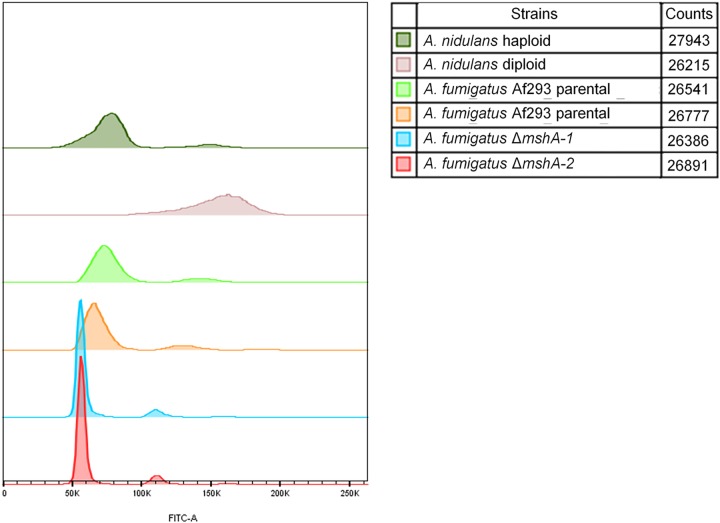
Fluorescence-activated cell sorting (FACS) analysis of A. nidulans and A. fumigatus DNA content.

**FIG 3:**
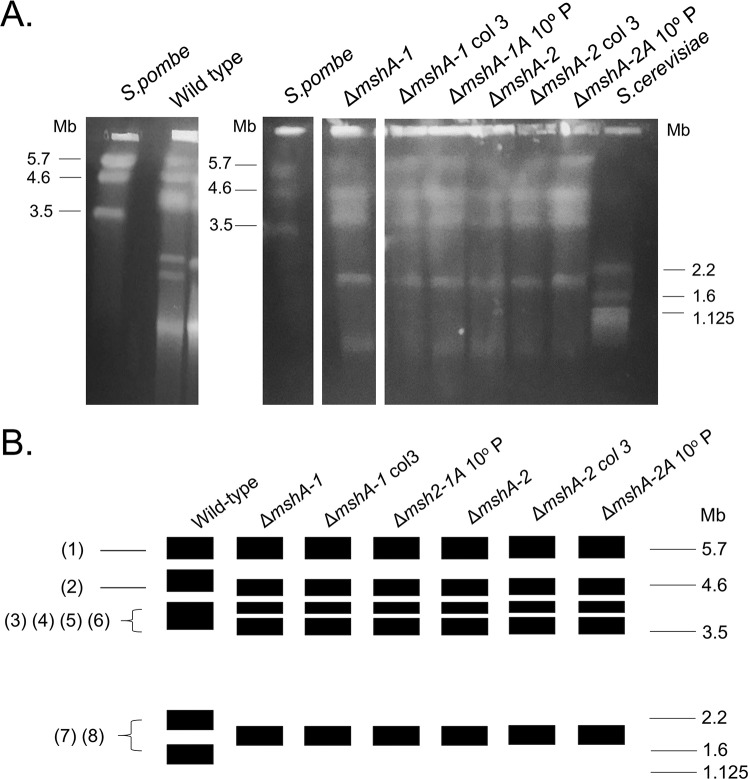
Karyotype polymorphism between wild-type strain and Δ*mshA* mutants of A. fumigatus. (A) Chromosomal bands separated by PFGE and stained with ethidium bromide. The gel on the left shows the chromosome profile of the A. fumigatus wild type. The profiles of the mutants were obtained in another gel shown at the right side of the figure panel (see the raw image in Fig. S2). The A. fumigatus wild type and mutants have five chromosomal bands. The smallest band (∼1.0 Mb) seen in the wild type and mutants might correspond to a minichromosome. S. pombe and S. cerevisiae chromosomal bands were used as size standards indicated in megabases (Mb). (B) Diagrammatic representation of karyotypes of the A. fumigatus wild type and mutants. The rectangles represent a unique distinguishable band visualized after staining with ethidium bromide. The thickness of the rectangles represents the proportional level of staining of each chromosomal band. The *in silico* chromosomes of A. fumigatus assigned to the chromosomal bands are indicated at the left.

We targeted *mshA* for deletion of the entire gene, aiming to further investigate its role in A. fumigatus (see Fig. S1 in the supplemental material). To eliminate the possibility of the occurrence of likely secondary mutations during the construction of deletion strains, we selected two independent transformants from each deletion experiment to pursue all our phenotypic analyses. First, we investigated whether the *mshA* null mutations had caused any effect on ploidy and chromosome (Chr) rearrangement (Fig. 2; see also Fig. 3). Fluorescence-activated cell sorting (FACS) analysis was used to compare the ploidy levels of the wild-type and mutant conidia (Fig. 2). Haploid and diploid strains of A. nidulans were used as controls for known cellular DNA content (Fig. 2). The Af293 wild-type parental and Δ*mshA-1* and Δ*mshA-2* mutant strains showed levels of DNA content consistent with a haploid distribution pattern, suggesting that the *mshA* mutations had not affected the ploidy (Fig. 2).

10.1128/mSphere.00416-19.1 FIG S1 Southern blotting strategy to confirm the deletion of Afu3g09850 (*mshA*) in A. fumigatus. (A) Schematic strategy for Southern blot analysis designed for confirmation of the *ΔmshA* deletion. (B) Genomic DNA was isolated from A. fumigatus wild-type and *ΔmshA* candidates and cleaved with the restriction enzyme BglI; a 1-kb DNA fragment from the 3′ noncoding region was used as a hybridization probe. This fragment recognizes a single DNA band (about 3.4 kb) in the wild-type strain and a single DNA band (about 4.1 kb) in the Δ*mshA* null mutant. Download FIG S1, PDF file, 0.1 MB. Copyright © 2019 dos Reis et al. 2019 dos Reis et al. This content is distributed under the terms of the Creative Commons Attribution 4.0 International license.

10.1128/mSphere.00416-19.2 FIG S2 Pulsed-field gel electrophoresis for the A. fumigatus wild type and mutants. Download FIG S2, PDF file, 0.1 MB. Copyright © 2019 dos Reis et al. 2019 dos Reis et al. This content is distributed under the terms of the Creative Commons Attribution 4.0 International license.

As previously described (19), the karyotype of the A. fumigatus wild type is composed of five chromosome-sized bands distributed as follows: three megabase bands of 5.7, 4.9, and 3.9 Mb; two middle-sized bands of 2.2 and 1.8 Mb (Fig. 3). The karyotype of mutants showed numerical and length-related chromosome polymorphisms comprising 4 megabase bands; the largest ones were of the same length (5.7 Mb) as the wild type, 3 others were slightly shorter (4.6, 4.1, and 3.6 Mb), and one had a middle-sized band of length 2.0 Mb. There was little (∼400 kb) variation in chromosome size between the wild type and mutants. The gross chromosomal rearrangements may have been the result of recombination events such as deletion, segmental duplication, and interchromosome fusion-fission. For instance, the 4.6-Mb band found in the mutants may have been generated from an ∼300-kb deletion in the 4.9-Mb band of the wild type. The loss of 2.2-Mb and 1.8-Mb bands and the simultaneous appearance of the 2.0-Mb band in the mutants may be explainable by events of deletion and segmental duplication in the 2.2-Mb and 1.8-Mb bands, respectively. Another explanation could be that fusion of the chromosomes occurred following fission into two smaller chromosomes of the same size (2.0 Mb). The chromosomal band of ∼1.0 Mb detected in all of the isolates may be related to minichromosomes described previously in strains and progeny of crosses of several fungal species (21).

We have assigned the *in silico* chromosomes (Chr1 to Chr8) of A. fumigatus Af293 to the chromosomal bands separated by pulsed-field gel electrophoresis (PFGE) (19). In the wild type, each chromosomal band is associated with only one chromosome, with the exception of the 3.9-Mb band, which harbors four *in silico* chromosomes (Chr3, Chr4, Chr5, and Chr6). In the *mshA* mutants, chromosomes 1 and 2 were assigned to the bands of 5.7 Mb and 4.6 Mb, respectively; Chr3, Chr4, and Chr5 to the 4.1-M band; Chr6 to 3.6-Mb band; and Chr7 and Chr8 to a single band of 2.0 Mb. Comparison of the karyotypic profiles of the wild type and mutants suggests that most of the chromosomal rearrangements were related to DNA loss. The genome of the mutants, estimated on the basis of the size of the chromosomal bands, is 11.4% smaller than that of the wild type, which indicates a loss of 3.3 Mb. It remains to be determined if this loss is restricted to only some areas of the genome, such as subtelomeric regions. Resequencing of mutants and mapping back to the wild type could address this issue. In agreement with the FACS analysis, these results strongly suggest that chromosomal rearrangements found in null mutants did not affect the ploidy, with a small reduction of DNA content in the mutants.

**FIG 4:**
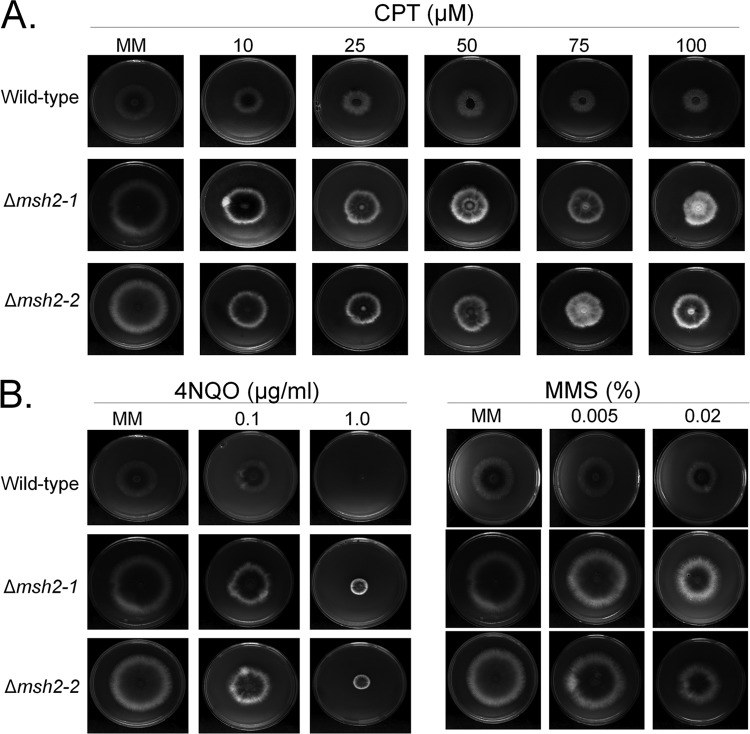
A. fumigatus Δ*mshA* mutants are not sensitive to DNA-damaging agents. A. fumigatus wild-type and Δ*mshA* conidia (1 × 10^4^) were inoculated on MM plus different drug concentrations. Plates were incubated for 5 days at 37°C. (A) CPT (camptothecin). (B) 4-NQO (4-nitroquinoline oxide). (C) MMS (methyl methanesulfonate).

We evaluated the impact of several DNA-damaging agents on the growth of the wild-type and mutant strains (Fig. 4). The *mshA* mutants showed the same resistance to CPT and MMS as the wild-type strain (Fig. 4), but they were more resistant to 4-NQO than the wild-type strain (Fig. 4B).

### *In vivo* analysis of the influence of A. fumigatus Δ*mshA* on virulence.

**FIG 5:**
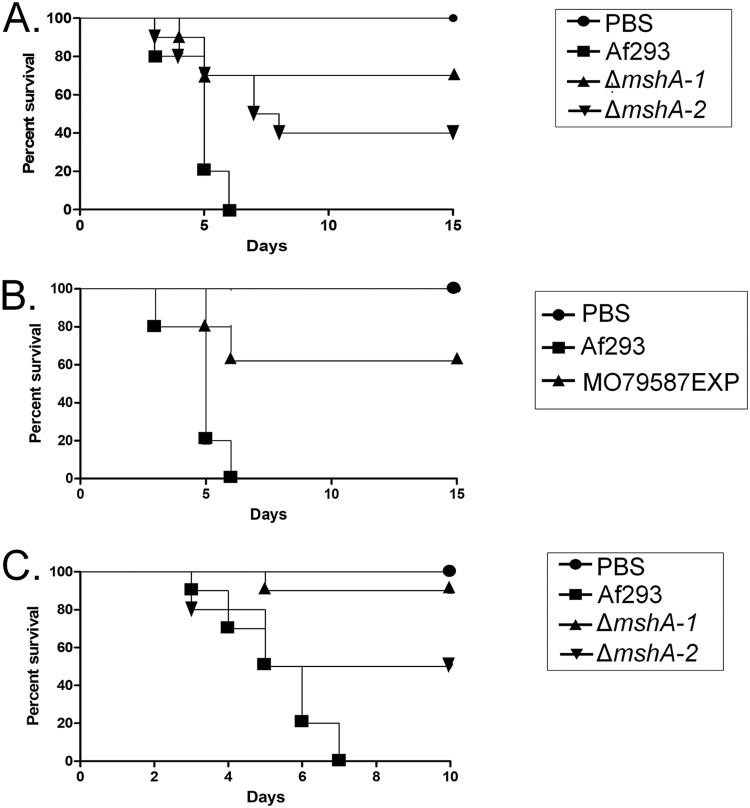
A. fumigatus *mshA* mutants have attenuated virulence in both a nutropenic mouse model and G. mellonella. (A and B) Comparative analysis of wild-type and mutant strains in a neutropenic murine model of pulmonary aspergillosis. Mice in groups of 10 per strain were infected intranasally with a 20-μl suspension of conidia at a dose of 10^5^ conidia. (A) Percent survival of Δ*mshA-1* and Δ*mshA-2* mutants compared to the wild-type Af293 strain. (B) Percent survival of MO79587EXP clinical isolate compared to the Af293 strain. (C) Comparative analysis of Af293 and *mshA* null mutants in G. mellonella animal model. Larvae in groups of 10 per strain were infected with a 5-μl suspension of conidia at a dose of 1 × 10^6^/larva. PBS, phosphate-buffered saline.

In the neutropenic murine model of IA, infection by the wild type strain, mutant Δ*mshA-1*, and mutant Δ*mshA-2* resulted in 90%, 30%, and 60% mortality 15 days postinfection, respectively (Fig. 5A). The levels of Δ*mshA-1* and Δ*mshA-2* virulence were attenuated compared to the levels seen with the wild-type strain according to the Mantel-Cox and Gehan-Brestow-Wilcoxon tests (Fig. 5A; *P* values of <0.05). We also tested the virulence of the MO79587EXP clinical isolate (which harbors two point mutations corresponding to Ala45Thr and Pro329Thr; Fig. 1C) in comparison to that of the Af293 strain (Fig. 5B). The MO79587EXP clinical isolate showed significant reduced virulence according to the Mantel-Cox and Gehan-Brestow-Wilcoxon tests (*P* values of <0.05) compared to another clinical isolate, namely, the Af293 strain (100% and 40% mortality, 7 and 16 days postinfection, for Af293 and in MO79587EXP, respectively) (Fig. 5B).

In another set of experiments, we used the moth Galleria mellonella as an alternative animal model to compare the levels of virulence of the mutants and the wild type. Both the Δ*mshA-1* and Δ*mshA-2* mutants showed significantly reduced virulence according to the Mantel-Cox and Gehan-Brestow-Wilcoxon tests (*P* values of <0.05) compared to the wild-type strain (100%, 10%, and 50% mortality 7 and 10 days postinfection for the wild-type strain, Δ*mshA-1* mutant, and Δ*mshA-2* mutant, respectively; Fig. 5C). These data suggest that, compared to the wild-type strain, the lack of mutations in *mshA* could influence virulence in A. fumigatus.

**FIG 6:**
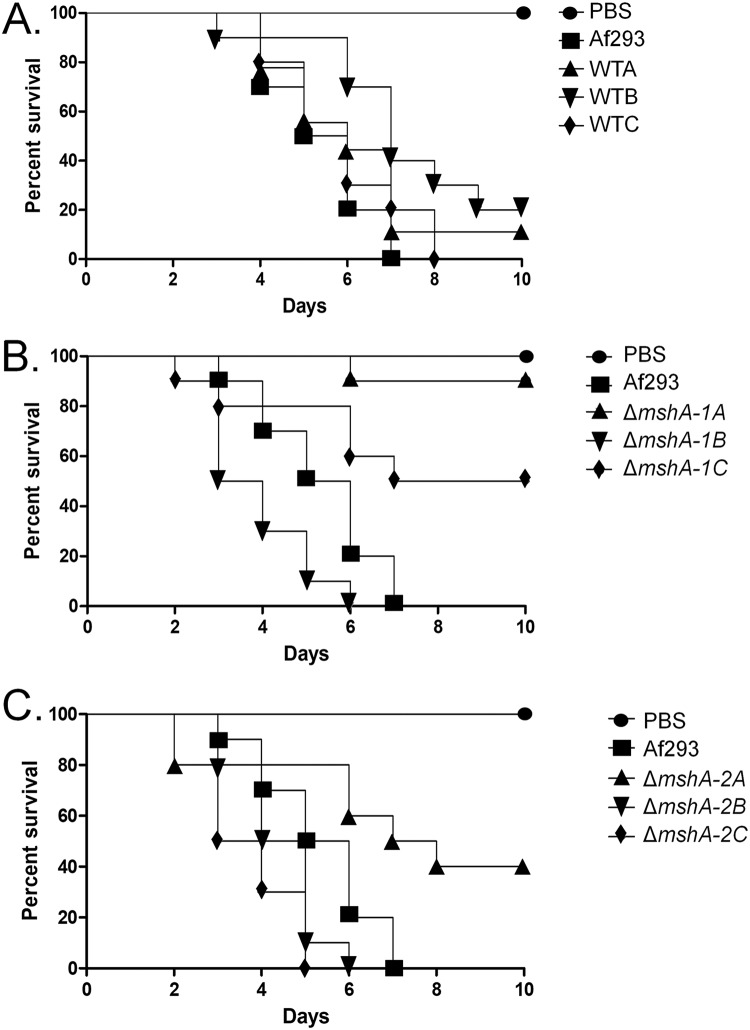
The Δ*mshA* mutants recovered their virulence after several rounds of mitotic division. Results of comparative analysis of the Af293, WTA-C, Δ*mshA-1A* to Δ*mshA-1C*, and Δ*mshA-2A* to Δ*mshA-2C* strains in the G. mellonella animal model are shown. Larvae in groups of 10 per strain were infected with a 5-μl suspension of conidia at a dose of 1 × 10^6^/larva. PBS, phosphate-buffered saline.

We verified whether sequential mitotic divisions of the wild-type strain and the Δ*mshA-1* and Δ*mshA-2* mutant strains without selective pressure would have an impact on virulence. First, we established three independent populations for each strain (wild-type A [WTA], WTB, and WTC populations; mutant ΔMSHA-1A, ΔMSHA-1B, and ΔMSHA-1C populations; and mutant ΔMSHA-2A, ΔMSHA-2B, and ΔMSHA-2C populations). We then transferred these populations through 10 conidial passages from each strain, except the parental ones, on MM plates and grew the populations at 37°C without any selective pressure. These evolved strains have shown the same phenotypes related to growth, conidiation, and sensitivity to DNA damage agents as those from the original parental strains (data not shown). We also have not observed major chromosomal differences among mutant Δ*mshA-1*, mutant Δ*mshA-2*, and 10th-passage strains Δ*msh-1A* and Δ*msh-2A* (Fig. 3). Conidia of the wild-type strain and the corresponding last-transferred populations were compared with respect to virulence by inoculating them in G. mellonella larvae. In the G. mellonella model, infection of all the strains resulted in 80% to 100% mortality at 7 to 10 days postinfection (Fig. 6A). There were no statistically significant differences among the strains (Mantel-Cox and Gehan-Brestow-Wilcoxon, *P* values of >0.05). Among the members of the ΔMSHA-1A, ΔMSHA-B, and ΔMSHA-C populations and the ΔMSHA-2A, ΔMSHA-B, and ΔMSHA-C populations, only members of the ΔMSHA-1A and ΔMSHA-2A populations were shown to be virulent (with levels that were not significantly different from those measured for the wild-type strain according to the Mantel-Cox and Gehan-Brestow-Wilcoxon tests, *P* values of >0.05) (Fig. 6B and C). These results strongly indicate that the genetic instability caused by the absence of MshA in A. fumigatus can evolve its virulence attributes in the G. mellonella model of infection.

### The influence of A. fumigatus Δ*mshA* mutants on azole resistance.

To examine if the lack of MshA could have any effect on azole resistance, we determined the MICs for voriconazole and posaconazole in the A. fumigatus wild-type, Δ*mshA-1*, and Δ*mshA-2* strains and found that they were 0.75 and 0.4 μg/ml, respectively (Table 2). The MEC (minimal effective concentration) for caspofungin for all those strains was 0.125 μg/ml (Table 2). Subsequently, we measured the posaconazole, voriconazole, and caspofungin MICs and MECs in the populations that had previously been transferred 10 times in MM (Table 2). Only populations Δ*mshA*-*1B* and Δ*mshA*-*1C* showed an increased voriconazole MIC (1.0 μg/ml).

**TABLE 2:** Drug MICs for A. fumigatus isolates from populations A, B, and C

Isolate	MIC (μg/ml)		
	Voriconazole	Posaconazole	Caspofungin
A. fumigatus WT (Af293)	0.75	0.4	0.125
A. fumigatus WT A	0.75	0.4	0.125
A. fumigatus WT B	0.75	0.4	0.125
A. fumigatus WT C	0.75	0.4	0.125
A. fumigatus Δ*mshA-1*	0.75	0.4	0.125
A. fumigatus Δ*mshA-1* A	0.75	0.4	0.125
A. fumigatus Δ*mshA-1* B	1	0.4	0.125
A. fumigatus Δ*mshA-1* C	1	0.4	0.125
A. fumigatus Δ*mshA-2*	0.75	0.4	0.125
A. fumigatus Δ*mshA-2* A	0.75	0.4	0.125
A. fumigatus Δ*mshA-2* B	0.75	0.4	0.125
A. fumigatus Δ*mshA-2* C	0.75	0.4	0.125

**FIG 7:**
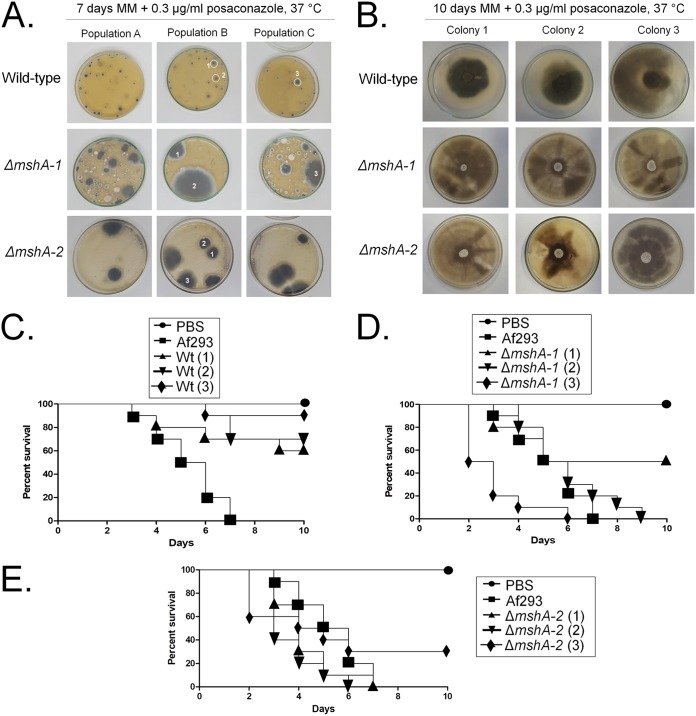
The wild-type and Δ*mshA* posaconazole-resistant mutants. (A) Conidia (1 × 10^8^) from the three original (without transfer) populations (populations A, B, and C) for each strain grown on MM plus 0.3 μg/ml of posaconazole for 7 days at 37°C. (B) Isolated mutants from each population prepared as described for panel A were subjected to point inoculation on MM plus 0.3 μg/ml posaconazole for 10 days at 37°C. (C to E) Comparative analysis of Af293 and the *ΔmshA* and posaconazole-resistant mutants in the G. mellonella animal model. (C) Wild-type strains. (D) Δ*mshA-1* strains. (E) Δ*mshA-2* strains. Larvae in groups of 10 per strain were infected with a 5-μl suspension of conidia at a dose of 1 × 10^6^/larva. PBS, phosphate-buffered saline.

We also plated 10^8^ conidia of the three original (without transfer) populations, i.e., populations A, B, and C, for each strain on MM plus 0.3 μg/ml of posaconazole for 7 days at 37°C (Fig. 7A). Several colonies grew in each plate, and we isolated three colonies from different populations (from the wild-type strain, two and one colonies from populations B and C, respectively; from the Δ*mshA-1* mutant, two colonies and one colony from populations B and C, respectively; from the Δ*mshA-2* mutant, three colonies from population B) (Fig. 7A). These colonies were streaked once on MM plus 0.3 μg/ml of posaconazole and centrally inoculated again on MM plus 0.3 μg/ml of posaconazole and allowed to grow for 10 days at 37°C. The colonies derived from the Δ*mshA-1* and Δ*mshA-2* strains showed a much greater number of sectors than those colonies derived from the wild-type strain, indicating their increased genetic instability (Fig. 7B). Nevertheless, we also did not observe major chromosomal differences among the Δ*mshA-1* mutant, colony 3 derived from the Δ*mshA-1* mutant, and colony 3 derived from the Δ*msh-2* mutant (Fig. 3). These strains were also much more resistant to posaconazole, but to not voriconazole and caspofungin, than their parental strains (Table 3). However, the strains derived from the Δ*mshA-1* and Δ*mshA-2* mutant strains were found to be at least 200-fold more resistant than those derived from the wild-type strain (Table 3). These results strongly indicate that Msh2 plays an important role in A. fumigatus genetic stability that dramatically affects its drug resistance.

**TABLE 3:** Drug MICs for A. fumigatus isolates from colonies 1, 2, and 3

Isolate	MIC (μg/ml)		
	Voriconazole	Posaconazole	Caspofungin
WT (Af293)	0.75	0.4	0.125
WT colony 1	1	2	0.125
WT colony 2	1	2	0.125
WT colony 3	1	2	0.125
Δ*mshA-1*	0.75	0.4	0.125
Δ*mshA-1* colony 1	0.75	>400	0.125
Δ*mshA-1* colony 2	0.75	>400	0.125
Δ*mshA-1* colony 3	0.75	>400	0.125
Δ*mshA-2*	0.75	0.4	0.125
Δ*mshA-2* colony 1	0.75	>400	0.125
Δ*mshA-2* colony 2	0.75	>400	0.125
Δ*mshA-2* colony 3	0.75	>400	0.125

We also evaluated the effect of the selection of these strains on G. mellonella virulence (Fig. 7C to E). The three colonies derived from the wild-type strain were found to be less virulent than the wild-type strain (Fig. 7C). In contrast, colonies 2 and 3 derived from the Δ*mshA-1* mutant became more virulent than the parental strain (and even more virulent than the wild-type strain for colony 3) (Fig. 7D) and colonies 1 and 2 derived from the Δ*mshA-2* strain became more virulent than the parental strain (Fig. 7E).

Taken together, our results strongly suggest that Msh2 plays an important role for A. fumigatus genetic stability and can dramatically impact drug resistance and virulence of this pathogen.

## DISCUSSION

MMR is a system for the correction of errors in which an incorrect base is incorporated into the daughter strand (22). MMR is essential for genomic stability, promoting genomic fidelity by repairing base-base mismatches, insertion deletion loops, and heterologies generated during DNA replication and recombination (22). S. cerevisiae Msh2p recognizes DNA mismatches forming heterodimers with Msh3p and Msh6p initiating the mismatch repair (23). The genomic instability favors the evolution of drug resistance in the presence of the selecting conditions such as in the clinical environment, as has already been observed for several fungal species (12–15). Here, we investigated the importance of MMR in A. fumigatus by screening a collection of environmental and clinical isolates for mutations in *MSH2* (here called *mshA*) and constructing *mshA* deletion strains and assessing their role in drug resistance and virulence.

We observed *mshA* variants in 18.2% of clinical and environmental A. fumigatus isolates investigated, which contrasts with the 55% MSH2 variants in C. glabrata clinical isolates reported previously (14). Interestingly, we observed three conserved spots of variants in *mshA* that impact three different MshA domains. Phenotypic characterization of one of these strains, MO79587EXP, which had had its genome previously sequenced by our group (20), suggested that this strain’s phenotypic profile is comparable to that of the Af293 clinical isolate but that it has reduced virulence compared to Af293, suggesting that *mshA* mutations could affect virulence. Actually, A. fumigatus *mshA* null mutants have reduced virulence compared to their parental Af293 strain. These results contrast with those determined for C. glabrata and C. neoformans *MSH2* null mutants, which demonstrated levels of colonization and infection similar to those seen with the corresponding wild-type strains (12, 13).

We grew the wild-type and *mshA* null mutants through 10 successive transfers on solid medium. The rationale behind this experiment was to provide an opportunity for the accumulation of mutations that could interfere with virulence and drug resistance. There were no dramatic changes in the virulence and drug resistance profiles of three independent populations of the wild-type strain. Interestingly, when the *mshA* null mutants were transferred onto solid medium, some of the independent populations gained virulence compared with the original null mutant. Considering that most of the DNA damage effects observed when MMR genes are lacking are related to the accumulation of nucleotide mutations, these results suggest that MMR defects in a fungal population could be important drivers of the emergence of more-virulent strains. Curiously, this was not observed with respect to drug resistance with either the wild-type or null mutants, suggesting that the occurrence of *MSH2* mutations in clinical isolates would favor the evolution of drug resistance under the conditions of strong drug selection normally present in the clinical environment. This is dramatically exemplified by the identification of azole-resistant mutants with high levels of posaconazole resistance when the *mshA* null mutants were subjected to a single round of voriconazole exposure. Although we had also isolated posaconazole-resistant mutants derived from the wild-type strains, those mutants were about 200-fold less sensitive to posaconazole than *mshA* null mutants. Surprisingly, the posaconazole-resistant mutants derived from the wild-type strain lost their virulence whereas some of those derived from *mshA* null mutants had gained virulence. Taken together, these results emphasize the importance of *MSH2* mutator phenotypes in the fitness of and in the establishment of virulence of A. fumigatus.

Our results provide significant information about the influence of *MSH2* mutations on drug resistance and virulence in A. fumigatus. Additional work will focus on the nature and the phenotypic effects of the mutations accumulated in these mutants that allow A. fumigatus to gain drug resistance and increased virulence.

## MATERIALS AND METHODS

### Ethics statement.

The principles that guide our studies are based on the Declaration of Animal Rights ratified by UNESCO (articles 8 and 14) on 27 January 1978. All protocols adopted in this study were approved by the local ethics committee for animal experiments from the University of São Paulo, Campus of Ribeirao Preto (permit 08.1.1277.53.6 [Studies on the interaction of Aspergillus fumigatus with animals]). Groups of five animals were housed in individually ventilated cages and were cared for in strict accordance with the principles outlined by the Brazilian College of Animal Experimentation (COBEA) and Guiding Principles for Research Involving Animals and Human Beings, American Physiological Society. All efforts were made to minimize suffering. Animals were clinically monitored at least twice daily and humanely sacrificed if moribund (defined by lethargy, dyspnea, hypothermia, and weight loss). All stressed animals were sacrificed by cervical dislocation.

### Strains, media, and growth conditions.

The A. fumigatus parental strain used in this study was strain Af293. All the constructed mutants were grown at 37°C in either minimal medium (MM; 1% glucose, 50 ml of a 20× salt solution [120 g/liter NaNO_3_, 10.4 g/liter KCl, 30 g/liter KH_2_PO_4_, 10.4 g/liter MgSO_4_] and 1 ml/liter of trace elements, pH 6.5) (24) or complete (yeast extract-glucose) medium {YG; 2% glucose, 0.5% yeast extract, and 1 ml/liter of trace elements [22.0 g/liter ZnSO_4_, 11 g/liter boric acid, 5 g/liter MnCl_2_, 5 g/liter FeSO_4_, 1.6 g/liter CoCl_2_, 1.6 g/liter CuSO_4_, 1.1 g/liter (NH_4_)_2_MoO_4_, 50 g/liter ethylenediaminetetraacetic acid]}. The media were made with and without 1% agar in order to obtain the solid medium and liquid medium, respectively. Additionally, uridine and uracil (1.2 g/liter each) were added as a nutritional supplement when necessary, resulting in YUU (YG plus UU) and MM plus UU media, respectively. A list of the strains used in this study is provided in Table S2 in the supplemental material.

10.1128/mSphere.00416-19.4 TABLE S2 Strains and plasmids used in this study. Download Table S2, DOCX file, 0.01 MB. Copyright © 2019 dos Reis et al. 2019 dos Reis et al. This content is distributed under the terms of the Creative Commons Attribution 4.0 International license.

### Identification of the A. fumigatus *MSH2* homolog and construction of the *Δmsh2* deletion mutant.

The A. fumigatus *MSH2* gene was deleted through the gene replacement approach, and the target gene was replaced with the *pyrG* prototrophic marker gene. The gene replacement cassette was constructed by *in vivo* recombination in Saccharomyces cerevisiae (25). Briefly, the genomic DNA (gDNA) of the Af293 strain was used as a template to PCR amplify ∼1 kb of the 5′-UTR and ∼1 kb of the 3′-UTR flanking regions of the MSH2 gene, and those fragments were amplified using specific primer pairs P1/P2 and P3/P4, respectively (Table S3). In addition, the *pyrG* prototrophic marker was amplified from pCDA21 plasmid (primers P5 and P6) (Table S3). After PCR amplification and DNA purification (from an agarose gel), the individual DNA fragments (the 5´UTR fragment, the 3´UTR fragment, and the *pyrG* marker fragment) were cotransformed with the linearized BamHI/EcoRI pRS426 plasmid (into S. cerevisiae strain FGSC SC9721) by the use of the lithium acetate method (25). The recombinant yeast candidates were selected in solid YNB-URA medium (7 g/liter yeast nitrogen base without amino acids, 0.05 g/liter histidine, 0.1 g/liter lysine, 0.1 g/liter leucine, 0.1 g/liter tryptophan, and 2% agar). In addition, the gDNA of the yeast candidates was extracted, and the deletion cassettes were subjected to PCR amplification using the outermost primers (P1 and P4) (Table S3). The gene replacement cassette was transformed into the A. fumigatus Af293 *pyrG*-negative background strain (25, 26). All PCR amplifications were performed using Phusion High-Fidelity DNA polymerase (New England Biolabs) or TaKaRa *Ex Taq* DNA polymerase (Clontech). The A. fumigatus candidates that gave positive test results were purified in a selective medium without uridine/uracil, and the gDNA was extracted and checked by Southern blotting using an AlkPhos direct labeling and detection system (GE Healthcare Life Sciences), according to the protocol of the manufacturer. Two independent transformants showing homologue integration of the deletion cassette at the *msh2* locus were selected for further analysis.

10.1128/mSphere.00416-19.5 TABLE S3 Primers used in this study. Download Table S3, DOCX file, 0.01 MB. Copyright © 2019 dos Reis et al. 2019 dos Reis et al. This content is distributed under the terms of the Creative Commons Attribution 4.0 International license.

### Measurements of DNA content per cell.

Conidia were collected, centrifuged (13,000 rpm for 3 min), and washed with sterile 1× phosphate-buffered saline (PBS) (8 g NaCl, 0.2 g KCl, 1.44 g Na_2_HPO_4_, and 0.24 g KH_2_PO_4_ per liter of sterilized water). For cell staining, the protocol described previously by Almeida et al. (27) was followed with modifications. Overnight fixation with 70% ethanol (vol/vol) was carried out at 4°C. Following that step, conidia were harvested, washed, and suspended in 850 μl of sodium citrate buffer (50 mM sodium citrate; pH 7.5). Briefly, sonicated conidia were treated for 1 h at 50°C with RNase A (Invitrogen, Waltham, MA, USA) (0.50 mg/ml) and for 2 h at 50°C with proteinase K (Sigma-Aldrich, St. Louis, MO, USA) (1 mg/ml). Conidia were stained overnight with SYBR green (Invitrogen, Carlsbad, CA, USA) (10,000×) diluted 10-fold in Tris-EDTA (pH 8.0), at a concentration of 2% (vol/vol) at 4°C. Finally, Triton X-100 (Sigma-Aldrich) was added to samples to reach a final concentration of 0.25% (vol/vol). Stained conidia were analyzed in a FACS LSRII flow cytometer (Becton, Dickinson, NJ, USA) with a 488-nm-excitation laser. Signals from a minimum of 30,000 cells per sample were captured in the fluorescein isothiocyanate (FITC) channel (530 nm ± 30 nm) at a low flow rate of about 1,000 cells/s, and an acquisition protocol was defined to measure forward scatter (FSC) and side scatter (SSC) on a 4-decade logarithmic scale and green fluorescence (FITC) on a linear scale. FACS Diva was used as the acquisition software. Results were analyzed with FlowJo software, version 10 (Tree Star), and with Modfit LT software (Verity Software House, Topsham, ME).

### Pulsed-field gel electrophoresis (PFGE) running conditions.

The agarose blocks containing chromosomal DNA from A. fumigatus isolates (wild-type strain and mutant strains) were submitted to pulsed-field gel electrophoresis. Gene Navigator System (Pharmacia) was used under the conditions described by Sasaki et al. (28), with minor modifications. The gels were prepared with Seaken agarose (FMC Bioproducts) (1.1%)–1× TAE (Tris-acetate-EDTA) according to electrophoretic run protocol, with a duration of 168 h and a constant voltage of 42 V at 10°C. The best electrophoretic resolution was achieved with homogeneous pulses of 900 s for 24 h, 1,800 s for 24 h, 2,700 s for 48 h, 3,600 s for 48 h and 4,500 s for 24 h, and the results were subjected to interpolation. Schizosaccharomyces pombe and S. cerevisiae chromosomal DNA was used as the molecular size standard in each of the electrophoretic runs. After this process, the gel was incubated in ethidium bromide solution (0.5 μl/ml) for 30 min and photographed under UV light.

### Phenotypic characterization of the Δ*mshA* null mutant.

A total of 10^5^ conidia of each strain tested were grown in solid MM in presence or absence of various concentrations of camptothecin (CPT; 10 μM, 25 μM, 50 μM, 75 μM, and 100 μM), methyl methanesulfonate (MMS; 0.005% and 0.02%), and 4-nitroquinoline-1-oxide (4-NQO; 0.1 μg/ml and 1 μg/ml). The plates were incubated for 120 h at 37°C, and radial growth was measured. The radial growth in the presence of drugs of each strain was normalized to the growth of each strain in MM without any drug. All plates were grown in triplicate, and averages ± standard deviations (SD) of the data are plotted.

### MIC and minimal effective concentration (MEC) analysis.

The drug susceptibility of the indicated strains was assessed by using the MIC method or the minimal effective concentration (MEC) method (29). The experiments were done in sterile 96-well plates containing 200 μl of liquid MM (with or without added antifungal drugs) plus 1 × 10^4^ conidia/well. After 48 h of incubation at 37°C, the MICs of the assayed drugs were determined visually as a no-growth endpoint. The MEC of caspofungin was defined as the lowest drug concentration that led to the growth of small, rounded, compact microcolonies compared to the growth control (caspofungin-free MM). The different drugs were diluted in the following ranges: (i) caspofungin at 0 to 2 μg/ml, (ii) voriconazole at 0 to 2 μg/ml, and posaconazole at 0 to 400 μg/ml. Three repetitions were performed for each treatment.

### Virulence analysis of the *mshA* mutants. (i) Murine model.

The levels of virulence of the wild-type strain, the *Δmsh2* null mutants, and the MO79587EXP clinical isolate were assayed through a murine model of pulmonary aspergillosis using outbreed female mice (BALB/c strain; body weight, 20 to 22 g). Briefly, the animals were housed in ventilated cages containing five mice each. For survival curve analyses, the animal immunosuppression and infection were done according to a previously described protocol (30). The statistical significance of the comparative survival values was calculated using the Mantel-Cox test, Gehan-Brestow-Wilcoxon log rank analysis, and the Prism statistical analysis package.

### (ii) Insect model.

The levels of virulence of the wild-type strain and the *Δmsh2* null mutants and their different populations were also analyzed using the larvae of G. mellonella obtained by breeding adult moths (31). The larvae infection protocol was done according to the method reported previously by dos Reis et al. (19).

### Maintenance of genetic stability in the *mshA* mutants after sequential mitotic division.

For the sequential mitotic division performed in solid MM in the absence of stress, the wild-type strain and *ΔmshA* null mutants were divided into 3 independent populations each, resulting in a total of 9 different populations: the WTA, WTB, and WTC populations; the Δ*mshA*-1A, Δ*mshA*-1B, Δ*mshA*-1C populations; and Δ*mshA*-2A, Δ*mshA*-2B, and Δ*mshA*-2C populations. Then, a total of 1 × 10^8^ conidia from each of the strains listed above were plated in solid MM and incubated 72 h at 37°C. The spores were harvested in sterile water, washed twice, and plated again to reach a final concentration of 1 × 10^8^ spores in MM plates and were incubated for an additional 72 h at 37°C. The procedure described above was repeated 10 times for each strain. In the end of the process, a total of 9 evolved populations were obtained and used for further assays.

Putative gains of azole resistance were also assayed by plating populations A, B, and C of the three original strains (the wild-type strain and the *ΔmshA* null mutant strains without transfer) in solid MM supplemented with posaconazole. Briefly, the experimental design was developed as follows. A total of 10^8^ conidia of original populations A to C of the wild-type and *ΔmshA-1* and *ΔmshA-2* mutant strains were plated in a total of 9 plates in solid MM supplemented with 0.3 μg/ml. All 9 plates were incubated at 37°C for 7 days, and several resistant colonies emerged from them. Then, 3 colonies were selected from each strain. Specifically, two colonies from population B and one colony from population C were selected from the WT plates, two colonies from population B and 1 colony from population C were selected from the mutant Δ*mshA-1* plates, and, finally, three colonies from population B were selected from the mutant Δ*mshA-2* plates. All of the selected colonies were individually streaked in MM supplemented with 0.3 μg/ml of posaconazole to increase the number of conidia, and the resulting reaction mixtures were used for further assays.

### Data availability.

All strains constructed in this study are available upon request. All data necessary for confirming the conclusions presented in the article are represented fully within the article.

## References

[1] Brakhage AA 2005 Systemic fungal infections caused by *Aspergillus* species: epidemiology, infection process and virulence determinants. Curr Drug Targets 6:875–886. doi:10.2174/138945005774912717. 16375671

[2] Brown GD, Denning DW, Levitz SM 2012 Tackling human fungal infections. Science 336:647. doi:10.1126/science.1222236. 22582229

[3] Brown GD, Denning DW, Gow NA, Levitz SM, Netea MG, White TC 2012 Hidden killers: human fungal infections. Sci Transl Med 4:165rv13. doi:10.1126/scitranslmed.3004404. 23253612

[4] Lackner M, Lass-Flörl C 2013 Up-date on diagnostic strategies of invasive aspergillosis. Curr Pharm Des 19:3595–3614. doi:10.2174/13816128113199990323. 23278540

[5] Valiante V, Macheleidt J, Föge M, Brakhage AA 2015 The *Aspergillus fumigatus* cell wall integrity signaling pathway: drug target, compensatory pathways, and virulence. Front Microbiol 6:325. 25932027 10.3389/fmicb.2015.00325 PMC4399325

[6] Denning DW 2002 A new class of antifungal. J Antimicrob Chemother 49:889–891. doi:10.1093/jac/dkf045. 12039879

[7] Walsh TJ, Anaissie EJ, Denning DW, Herbrecht R, Kontoyiannis DP, Marr KA, Morrison VA, Segal BH, Steinbach WJ, Stevens DA, van Burik JA, Wingard JR, Patterson TF; Infectious Diseases Society of America. 2008 Treatment of aspergillosis: clinical practice guidelines of the Infectious Diseases Society of America. Clin Infect Dis 46:327–360. doi:10.1086/525258. 18177225

[8] Resendiz Sharpe A, Lagrou K, Meis JF, Chowdhary A, Lockhart SR, Verweij PE; ISHAM/ECMM Aspergillus Resistance Surveillance working group. 2018 Triazole resistance surveillance in Aspergillus fumigatus. Med Mycol 56:83–92. 29538741 10.1093/mmy/myx144 PMC11950814

[9] Hagiwara D, Watanabe A, Kamei K, Goldman GH 2016 Epidemiological and genomic landscape of azole resistance mechanisms in Aspergillus fungi. Front Microbiol 7:1382. doi:10.3389/fmicb.2016.01382. 27708619 PMC5030247

[10] Iyer R, Pluciennik A, Burdett V, Modrich P 2006 DNA mismatch repair: functions and mechanisms. Chem Rev 106:302–323. doi:10.1021/cr0404794. 16464007

[11] Larrea AA, Lujan SA, Kunkel TA 2010 DNA mismatch repair. Cell 141:730. doi:10.1016/j.cell.2010.05.002. 20478261

[12] Legrand M, Chan CL, Jauert PA, Kirkpatrick DT 2007 Role of DNA mismatch repair and double-strand break repair in genome stability and antifungal drug resistance in *Candida albicans*. Eukaryot Cell 6:2194–2205. doi:10.1128/EC.00299-07. 17965250 PMC2168241

[13] Boyce KJ, Wang Y, Verma S, Shakya VPS, Xue C, Idnurm A 2017 Mismatch repair of DNA replication errors contributes to microevolution in the pathogenic fungus *Cryptococcus neoformans*. mBio 8:e00595-17. doi:10.1128/mBio.00595-17. 28559486 PMC5449657

[14] Healey KR, Zhao Y, Perez WB, Lockhart SR, Sobel JD, Farmakiotis D, Kontoyiannis DP, Sanglard D, Taj-Aldeen SJ, Alexander BD, Jimenez-Ortigosa C, Shor E, Perlin DS 2016 Prevalent mutator genotype identified in fungal pathogen *Candida glabrata* promotes multi-drug resistance. Nat Commun 7:11128. doi:10.1038/ncomms11128. 27020939 PMC5603725

[15] Healey KR, Jimenez Ortigosa C, Shor E, Perlin DS 2016 Genetic drivers of multidrug resistance in *Candida glabrata*. Front Microbiol 7:1995. doi:10.3389/fmicb.2016.01995. 28018323 PMC5156712

[16] Dellière S, Healey K, Gits-Muselli M, Carrara B, Barbaro A, Guigue N, Lecefel C, Touratier S, Desnos-Ollivier M, Perlin DS, Bretagne S, Alanio A 2016 Fluconazole and echinocandin resistance of Candida glabrata correlates better with antifungal drug exposure rather than with MSH2 mutator genotype in a French cohort of patients harboring low rates of resistance. Front Microbiol 7:2038. 28066361 10.3389/fmicb.2016.02038 PMC5179511

[17] Singh A, Healey KR, Yadav P, Upadhyaya G, Sachdeva N, Sarma S, Kumar A, Tarai B, Perlin DS, Chowdhary A 2018 Absence of azole or echinocandin resistance in *Candida glabrata* isolates in India despite background prevalence of strains with defects in DNA mismatch repair pathway. Antimicrob Agents Chemother 62:e00195-18. doi:10.1128/AAC.00195-18. 29610199 PMC5971596

[18] Hou X, Xiao M, Wang H, Yu SY, Zhang G, Zhao Y, Xu YC 2018 Profiling of *PDR1* and *MSH2* in *Candida glabrata* bloodstream isolates from a multicenter study in China. Antimicrob Agents Chemother 62:e00153-18. doi:10.1128/AAC.00153-18. 29581110 PMC5971605

[19] dos Reis TF, Silva LP, de Castro PA, Almeida de Lima PB, do Carmo RA, Marini MM, da Silveira JF, Ferreira BH, Rodrigues F, Malavazi I, Goldman GH 2018 The influence of genetic stability on Aspergillus fumigatus virulence and azole resistance. G3 (Bethesda) 8:265–278. doi:10.1534/g3.117.300265. 29150592 PMC5765354

[20] Lind AL, Wisecaver JH, Lameiras C, Wiemann P, Palmer JM, Keller NP, Rodrigues F, Goldman GH, Rokas A 2017 Drivers of genetic diversity in secondary metabolic gene clusters within a fungal species. PLoS Biol 15:e2003583. doi:10.1371/journal.pbio.2003583. 29149178 PMC5711037

[21] Zolan ME 1995 Chromosome-length polymorphism in fungi. Microbiol Rev 59:686–698. 8531892 10.1128/mr.59.4.686-698.1995 PMC239395

[22] Gupta D, Heinen CD 2019 The mismatch repair-dependent DNA damage response: mechanisms and implications. DNA Repair (Amst) 78:60–69. doi:10.1016/j.dnarep.2019.03.009. 30959407

[23] Boiteux S, Jinks-Robertson S 2013 DNA repair mechanisms and the bypass of DNA damage in *Saccharomyces cerevisiae*. Genetics 193:1025–1064. doi:10.1534/genetics.112.145219. 23547164 PMC3606085

[24] Kafer E 1977 Meiotic and mitotic recombination in *Aspergillus* and its chromosomal aberrations. Adv Genet 19:33–131. doi:10.1016/S0065-2660(08)60245-X. 327767

[25] Malavazi I, Goldman GH 2012 Gene disruption in *Aspergillus fumigatus* using a PCR-based strategy and in vivo recombination in yeast. Methods Mol Biol 845:99–118. doi:10.1007/978-1-61779-539-8_7. 22328370

[26] Osmani SA, May GS, Morris NR 1987 Regulation of the mRNA levels of *nimA*, a gene required for the G2-M transition in *Aspergillus nidulans*. J Cell Biol 104:1495–1504. doi:10.1083/jcb.104.6.1495. 3294854 PMC2114495

[27] Almeida AJ, Matute DR, Carmona JA, Martins M, Torres I, McEwen JG, Restrepo A, Leão C, Ludovico P, Rodrigues F 2007 Genome size and ploidy of *Paracoccidioides brasiliensis* reveals a haploid DNA content: flow cytometry and GP43 sequence analysis. Fungal Genet Biol 44:25–31. doi:10.1016/j.fgb.2006.06.003. 16879998

[28] Sasaki AA, Fernandes GF, Rodrigues AM, Lima FM, Marini MM, Dos S Feitosa L, de Melo Teixeira M, Felipe MS, da Silveira JF, de Camargo ZP 2014 Chromosomal polymorphism in the Sporothrix schenckii complex. PLoS One 9:e86819. doi:10.1371/journal.pone.0086819. 24466257 PMC3900657

[29] CLSI. 2008 Reference method for broth dilution antifungal susceptibility testing of filamentous fungi, M38-A2, 2nd ed CLSI, Wayne, PA.

[30] Dinamarco TM, Almeida RS, de Castro PA, Brown NA, dos Reis TF, Ramalho LN, Savoldi M, Goldman MH, Goldman GH 2012 Molecular characterization of the putative transcription factor SebA involved in virulence in Aspergillus fumigatus. Eukaryot Cell 11:518–531. doi:10.1128/EC.00016-12. 22345349 PMC3318302

[31] Fuchs BB, O’Brien E, Khoury JB, Mylonakis E 2010 Methods for using *Galleria mellonella* as a model host to study fungal pathogenesis. Virulence 1:475–482. doi:10.4161/viru.1.6.12985. 21178491

