# Supplementary figures and images for: The Aspergillus fumigatus Mismatch Repair MSH2 Homolog Is Important for Virulence and Azole Resistance
Source: mSphere. 2019 Aug 7;4(4):e00416-19. doi: 10.1128/mSphere.00416-19 (PMC6686229; doi:10.1128/mSphere.00416-19)

# Southern Blot – $\Delta mshA$ null mutant (Afu3g09850)

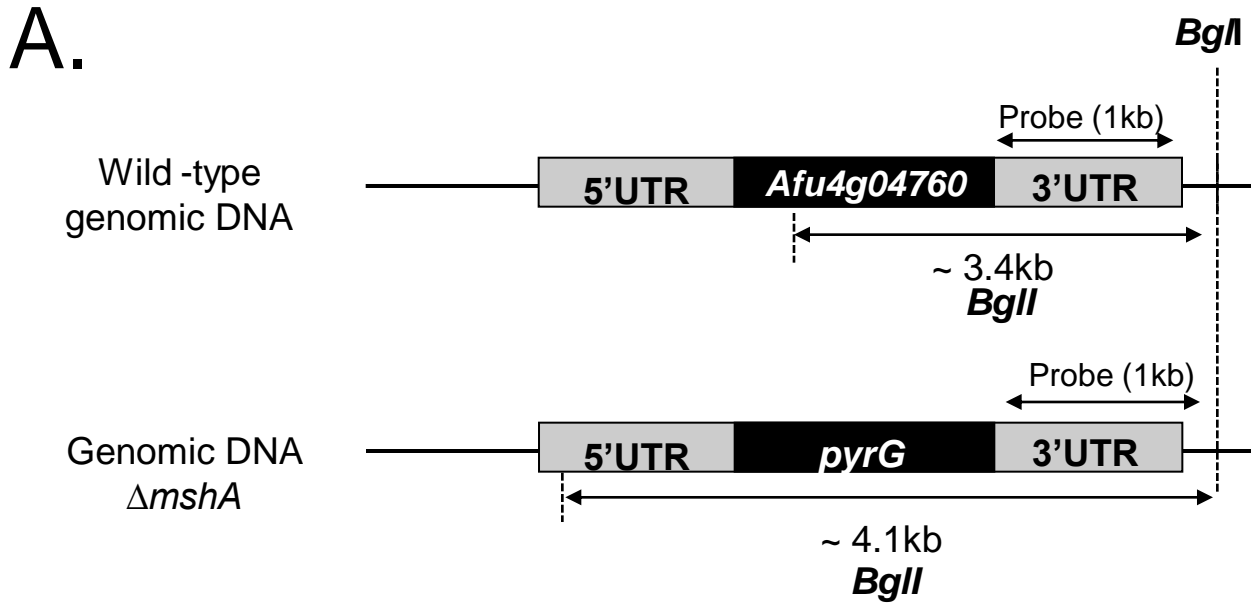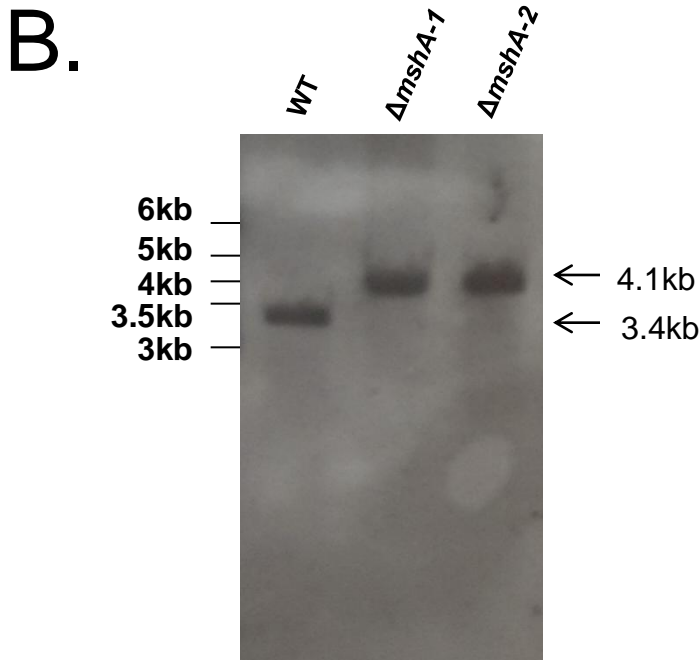

Supplement: FIG S1 [file mSphere.00416-19-sf001.pdf]
