# Supplementary material for: The Aspergillus fumigatus Mismatch Repair MSH2 Homolog Is Important for Virulence and Azole Resistance
Source: mSphere. 2019 Aug 7;4(4):e00416-19. doi: 10.1128/mSphere.00416-19 (PMC6686229; doi:10.1128/mSphere.00416-19)
Supplement: FIG S2 [file mSphere.00416-19-sf002.pdf]

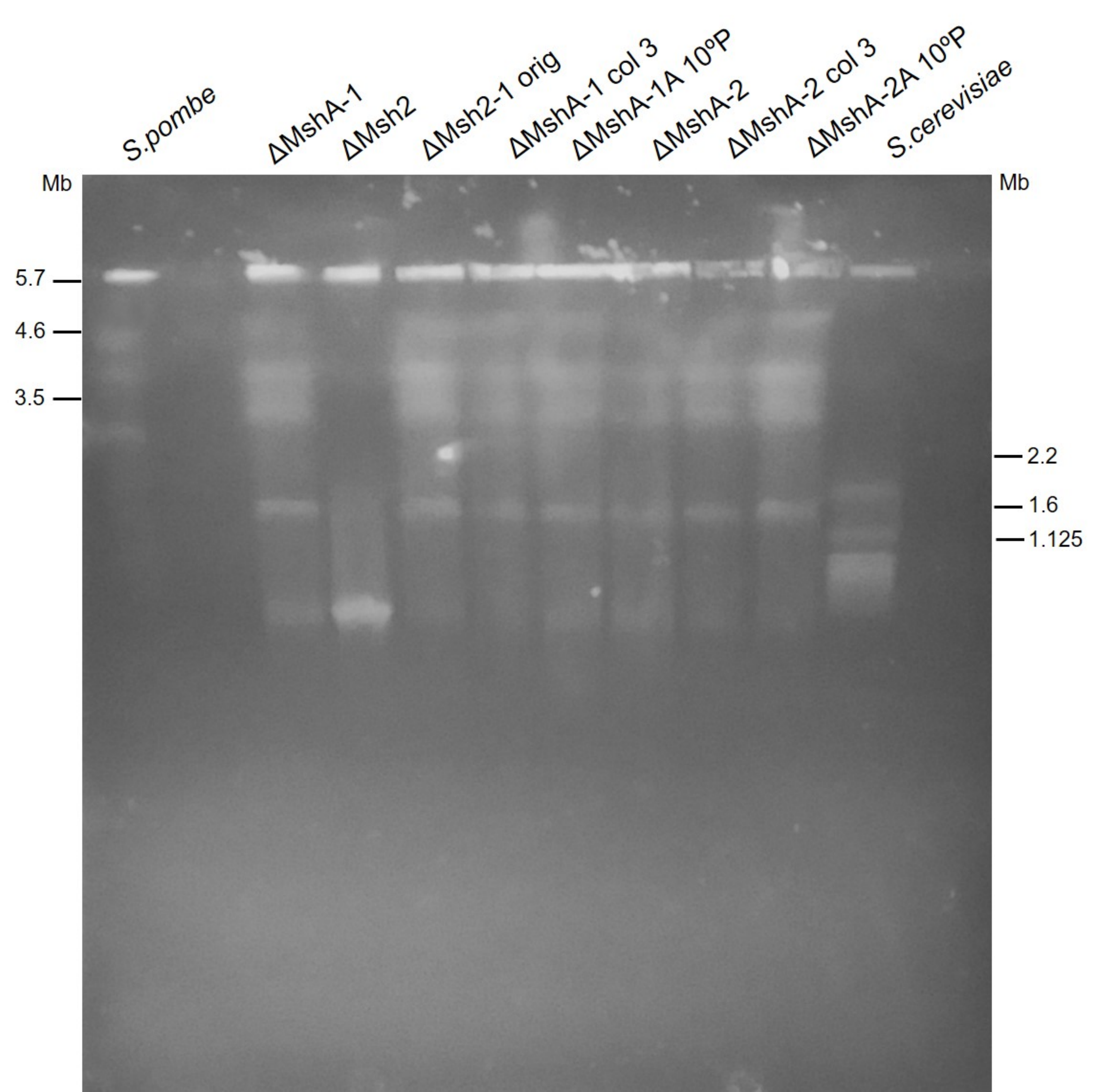
S.pombe
ΔMshA-1
ΔMsh2
ΔMsh2-1 orig
ΔMshA-1 col 3
ΔMshA-1A 10ºP
ΔMshA-2
ΔMshA-2 col 3
ΔMshA-2A 10ºP
S.cerevisiae
Mb
5.7
4.6
3.5
Mb
2.2
1.6
1.125
