# Supplementary material for: The Aspergillus fumigatus Mismatch Repair MSH2 Homolog Is Important for Virulence and Azole Resistance
Source: mSphere. 2019 Aug 7;4(4):e00416-19. doi: 10.1128/mSphere.00416-19 (PMC6686229; doi:10.1128/mSphere.00416-19)
Supplement: TABLE S2 [file mSphere.00416-19-st002.docx]

| **STRAINS/PLASMID** | **GENOTYPE/DESCRIPTION** | **REFERENCE** |
| --- | --- | --- |
| **Plasmid** |  |  |
| **pRS426** | *ampR lacZ* URA3 | Teepe *et al*., 2007 |
| ***S. cerevisiae*** |  |  |
| **SC9721** | MATa; his2D200; URA 3-52; leu2D1; lys2D202; trp1D63 | FGSC |
| ***A. fumigatus*** |  |  |
| **Af293** | pyrG- | FGSC |
| **MO79587EXP** | Clinical isolate: unknown genotype | Lind *et al*., 2017 |
| ***ΔmshA-1*** | pyrG; ΔmshA::pyrG | This study |
| ***ΔmshA*-2** | pyrG; ΔmshA::pyrG | This study |
| **WTA** | Population A generated from Af293 after 10 sequencial mitotic passages in MM without selective pressure | This study |
| **WTB** | Population B generated from Af293 after 10 sequencial mitotic passages in MM without selective pressure | This study |
| **WTC** | Population C generated from Af293 after 10 sequencial mitotic passages in MM without selective pressure | This study |
| **ΔmshA-1A** | Population A generated from ΔmshA-1 after 10 sequencial mitotic passages in MM without selective pressure | This study |
| **ΔmshA-1 B** | Population B generated from ΔmshA-1 after 10 sequencial mitotic passages in MM without selective pressure | This study |
| **ΔmshA-1C** | Population C generated from ΔmshA-1 after 10 sequencial mitotic passages in MM without selective pressure | This study |
| **ΔmshA-2A** | Population A generated from ΔmshA-2 after 10 sequencial mitotic passages in MM without selective pressure | This study |
| **ΔmshA-2B** | Population B generated from ΔmshA-2 after 10 sequencial mitotic passages in MM without selective pressure | This study |
| **ΔmshA-2C** | Population C generated from ΔmshA-2 after 10 sequencial mitotic passages in MM without selective pressure | This study |
| **WT colony A** | Colony A obtained from Af293 plated in MM supplemented with posaconazole | This study |
| **WT colony B** | Colony B obtained from Af293 plated in MM supplemented with posaconazole | This study |
| **WT colony C** | Colony C obtained from Af293 plated in MM supplemented with posaconazole | This study |
| **ΔmshA1- colony A** | Colony A obtained from ΔmshA-1plated in MM supplemented with posaconazole | This study |
| **ΔmshA-1 colony B** | Colony B obtained from ΔmshA-1plated in MM supplemented with posaconazole | This study |
| **ΔmshA-1 colony C** | Colony C obtained from ΔmshA-1plated in MM supplemented with posaconazole | This study |
| **ΔmshA-2 colony A** | Colony A obtained from ΔmshA-2 plated in MM supplemented with posaconazole | This study |
| **ΔmshA-2 colony B** | Colony B obtained from ΔmshA-2 plated in MM supplemented with posaconazole | This study |
| **ΔmshA-2 colony C** | Colony C obtained from ΔmshA-2 plated in MM supplemented with posaconazole | This study |
