# Supplementary material for: The Aspergillus fumigatus Mismatch Repair MSH2 Homolog Is Important for Virulence and Azole Resistance
Source: mSphere. 2019 Aug 7;4(4):e00416-19. doi: 10.1128/mSphere.00416-19 (PMC6686229; doi:10.1128/mSphere.00416-19)
Supplement: TABLE S3 [file mSphere.00416-19-st003.docx]

|  | **name** | **Sequence (5´- 3´)** |
| --- | --- | --- |
| P1 | mshA pRS426 5F | GTAACGCCAGGGTTTTCCCAGTCACGACGCTACGTTTCGAAAGGGCCAGCAGC |
| P2 | mshA pyrG 5R | CAGTGCCTCCTCTCAGACAGAATTGTGCTAGCGTTGCCCGATACAATG |
| P3 | mshA pyrG 3F | GAGCATTGTTTGAGGCGAATTCCTTCCACAAAGCTTCTTTATAAG |
| P4 | mshA pyrG 3R | GCGGATAACAATTTCACACAGGAAACAGCGAAGTAAATTTTAGTAGCAGAGG |
| P5 | pyrG F | ATTCTGTCTGAGAGGAGGCACTGATGCG |
| P6 | pyrG R | GAATTCGCCTCAAACAATGCTCTTCACC |
